# High-resolution habitat suitability model for *Phlebotomus pedifer*, the vector of cutaneous leishmaniasis in southwestern Ethiopia

**DOI:** 10.1186/s13071-020-04336-3

**Published:** 2020-09-11

**Authors:** Myrthe Pareyn, Anneleen Rutten, Behailu Merdekios, Ronja E. M. Wedegärtner, Nigatu Girma, Leo Regelbrugge, Simon Shibru, Herwig Leirs

**Affiliations:** 1 Evolutionary Ecology Group, University of Antwerp, Antwerp, Belgium; 2 Public Health Department, Arba Minch University, Arba Minch, Ethiopia; 3 Department of Biology, Norwegian University of Science and Technology, Trondheim, Norway; 4 Biology Department, Arba Minch University, Arba Minch, Ethiopia

**Keywords:** Species distribution modeling, Maximum entropy, Sand fly, *Phlebotomus pedifer*, Ethiopia, Topoclimate, Downscaling, Cutaneous leishmaniasis

## Abstract

**Background:**

*Phlebotomus pedifer* is the vector for *Leishmania aethiopica* causing cutaneous leishmaniasis (CL) in southwestern Ethiopia. Previous research on the transmission dynamics of CL resulted in recommendations for vector control. In order to target these interventions towards affected areas, a comprehensive understanding of the spatial distribution of *P. pedifer* at high spatial resolution is required. Therefore, this study determined the environmental predictors that facilitate the distribution of *P. pedifer* and created a map indicating the areas where conditions are suitable for survival of the vector in southwestern Ethiopia with high spatial resolution.

**Methods:**

*Phlebotomus pedifer* presence points were collected during two entomological surveys. Climate, vegetation and topographic variables were assembled. Climate variables were interpolated with variables derived from high-resolution digital elevation models to generate topoclimatic layers representing the climate conditions in the highlands. A Maximum Entropy model was run with the presence points, predicting variables and background points, which were selected based on a bias file.

**Results:**

*Phlebotomus pedifer* was the only captured *Phlebotomus* species in the study area and was collected at altitudes ranging between 1685 and 2892 m. Model projections indicated areas with suitable conditions in a ‘belt’ surrounding the high mountain peaks. Model performance was high, with train and test AUC values being 0.93 and 0.90, respectively. A multivariate environmental similarity surface (MESS) analysis showed that the model projection was only slightly extrapolated for some of the variables. The mean annual temperature was the environmental variable, which contributed most to the model predictions (60.0%) followed by the seasonality in rainfall (13.2%). Variables representing steep slopes showed very low importance to model predictions.

**Conclusions:**

Our findings indicate that the suitable habitats for *P. pedifer* correspond well with the altitudes at which CL was reported previously, but the predictions are more widely distributed, in contrast with the description of CL to occur in particular foci. Moreover, we confirm that vector distribution is driven by climate factors, suggesting inclusion of topoclimate in sand fly distribution models. Overall, our model provides a map with a high spatial resolution that can be used to target sand fly control measures in southwestern Ethiopia.
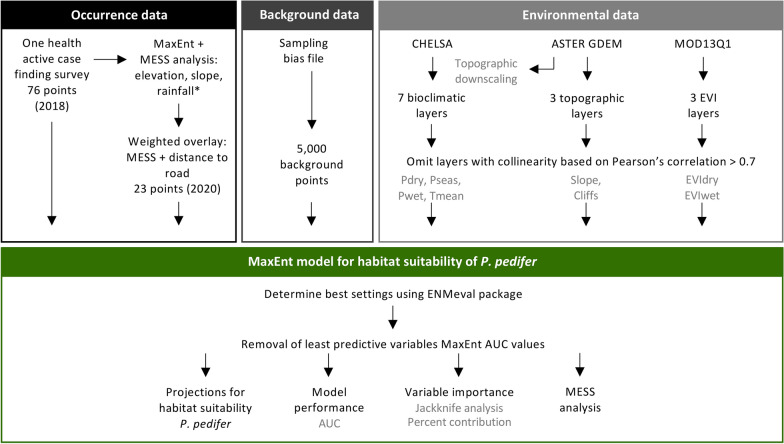

## Background

Phlebotomine sand flies (Diptera: Phlebotominae) are tiny, hematophagous insects that occur in tropical and subtropical regions. In Africa, the genera *Phlebotomus* and *Sergentomyia* occur and some species of the former genus are the vector of *Leishmania* spp., causing leishmaniasis in humans [1]. The infection can manifest in three major clinical forms: cutaneous (CL), mucocutaneous (MCL) and visceral (VL) leishmaniasis, which are all three occurring in Ethiopia [2–4]. The most common form is CL, which is caused by *Leishmania aethiopica*. This parasite species is transmitted by *Phlebotomus pedifer* Lewis, Mutinga & Ashford, 1972 in southwestern and *P. longipes* Parrot & Martin, 1939 in central and northern Ethiopia [5–7].

In great contrast to the 878 CL cases that were reported to the WHO in 2018, it is estimated that the incidence of the infection lies between 20,000–50,000 cases yearly, reflecting the severe underreporting of the infection in Ethiopia [4, 8]. CL particularly occurs in foci on the mountain slopes of the Ethiopian Rift Valley, ranging from North to Southwest and South to Northeast in the country. The described foci are all situated at altitudes ranging between 1700–2700 m and are located in four regional states: Southern Nations, Nationalities and Peoples’ Region (SNNPR), Amhara, Tigray and Oromia, and Addis Ababa city administration [5, 6, 9–13].

Ochollo is a well-known CL focus at about 2100 m in southwestern Ethiopia and is considered a model village for research investigating the transmission dynamics of CL [6, 9, 14]. The area has a rough topography and is characterized by steep slopes, many rocks and basalt cliffs with caves, providing the ideal habitat for *P. pedifer* and the animal reservoir of the infection, hyraxes [15]. According to findings on the transmission cycle in Ochollo, suggestions have been made for vector control and disease prevention in the area [6, 9, 14].

Effective and efficient implementation of integrated vector control programmes and resource allocation requires a comprehensive understanding of the spatial distribution of *P. pedifer*. Besides from Ochollo and an outbreak in Silte woreda, neither *P. pedifer* nor CL has been reported in the surrounding areas, even though the topography and ecology appear similar in some areas [6, 9, 11, 14, 15]. However, a recent study indicated many of these areas to be at high-risk for CL based on environmental parameters (rainfall, altitude and slope), yet no (entomological) surveys have been conducted here [16].

It is quite novel that species distribution models (SDMs) are being implemented to predict the distribution of a vector to optimize control measures [17]. SDMs are sophisticated, dynamic tools that can identify areas that are suitable for the survival of a particular species. It integrates species occurrence data and information about environmental conditions at these locations to characterize the niche of the species and project it into the geographical space, resulting in a map that predicts the species’ potential distribution [18, 19].

Commonly, bioclimatic variables are applied in SDMs at 30 arc-second resolution (1 km^2^) or coarser, which represent free-air conditions that were averaged over the past 30 years [20–22]. Although these layers are probably adequate for flat terrains, they may not be sufficient for representation in mountainous areas with a variable topography [23–26]. Due to this vertical dimension, organisms experience microclimatic conditions, which can vary noticeably over a short distance. This is attributed to several topographic factors, such as slope angle, aspect, solar radiation, distance to the ocean, etc. [27]. An additional issue of these macroclimatic data is that other layers with a higher spatial resolution need to be resampled, which can lead to loss of important details.

However, macroclimatic data can be downscaled with variables derived from high-resolution digital elevation models (DEMs) to generate a statistical relationship that results in higher resolution climatic data [23, 24]. Integration of these high-resolution climatic variables was demonstrated to significantly improve the predictive power of SDMs [28, 29].

In this study, we used topoclimatic variables in an SDM to determine the environmental predictors that facilitate the presence of *P. pedifer* and assessed areas that are suitable for the survival of the vector with high spatial resolution in five zones of the SNNPR. The generated maps can be implemented by policymakers for guidance of targeted vector control programs to reduce the burden of CL in this area in southwestern Ethiopia.

## Methods

### Site description

**Fig. 1:**
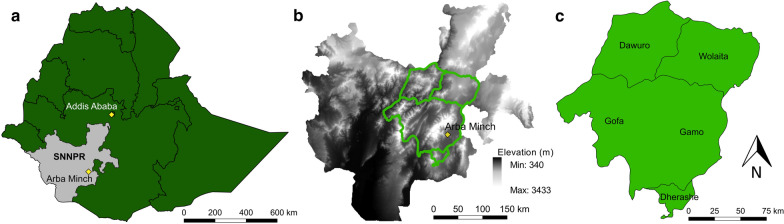
Location of the study area [78, 79]. **a** The SNNPR in southwestern Ethiopia, with the capital city Arba Minch situated in the east. **b** Magnification of the topography (elevation, metres) of the SNNPR and the study area indicated in green. **c** Magnification of the study area: four zones (Dawuro, Wolaita, Gofa and Gamo) and Dherashe area. *Abbreviation:* SNNPR, Southern Nations, Nationalities and Peoples’ Region

The study was conducted in the SNNPR, in southwestern Ethiopia (Fig. 1a). The area has a variable topography with an altitude ranging from 340 to 3433 m (Fig. 1b). The south and west of the area comprise flat lowlands, whereas the north and east are mountainous. *P. pedifer* occurs in mountainous areas and the model village for research on CL transmission, Ochollo, is situated near Arba Minch, in Gamo zone. Therefore, we selected Gamo zone, three additional surrounding administrative zones, including Gofa, Wolaita and Dawuro and Dherashe area for sample collection (Fig. 1c). Together, the area covers approximately 22,000 km^2^ and is inhabited by 4.7 million people.

The study area consists of mountains and valleys with an altitude ranging between 550 and 3390 m. Due to its topography, it has a temperate climate, with an average yearly temperature ranging from 9.3 °C to 25.5 °C and rainfall varying between 630 mm and 2280 mm, in the lowlands and highlands [30]. Because the study area covers a wide topographical range, the seasons vary from place to place, but generally the dry season lasts from October to April and the wet season from May to September. In recent years, the area has been subjected to ecological modifications related to human activities, like urbanization, agriculture and deforestation.

### Occurrence data

**Fig. 2:**
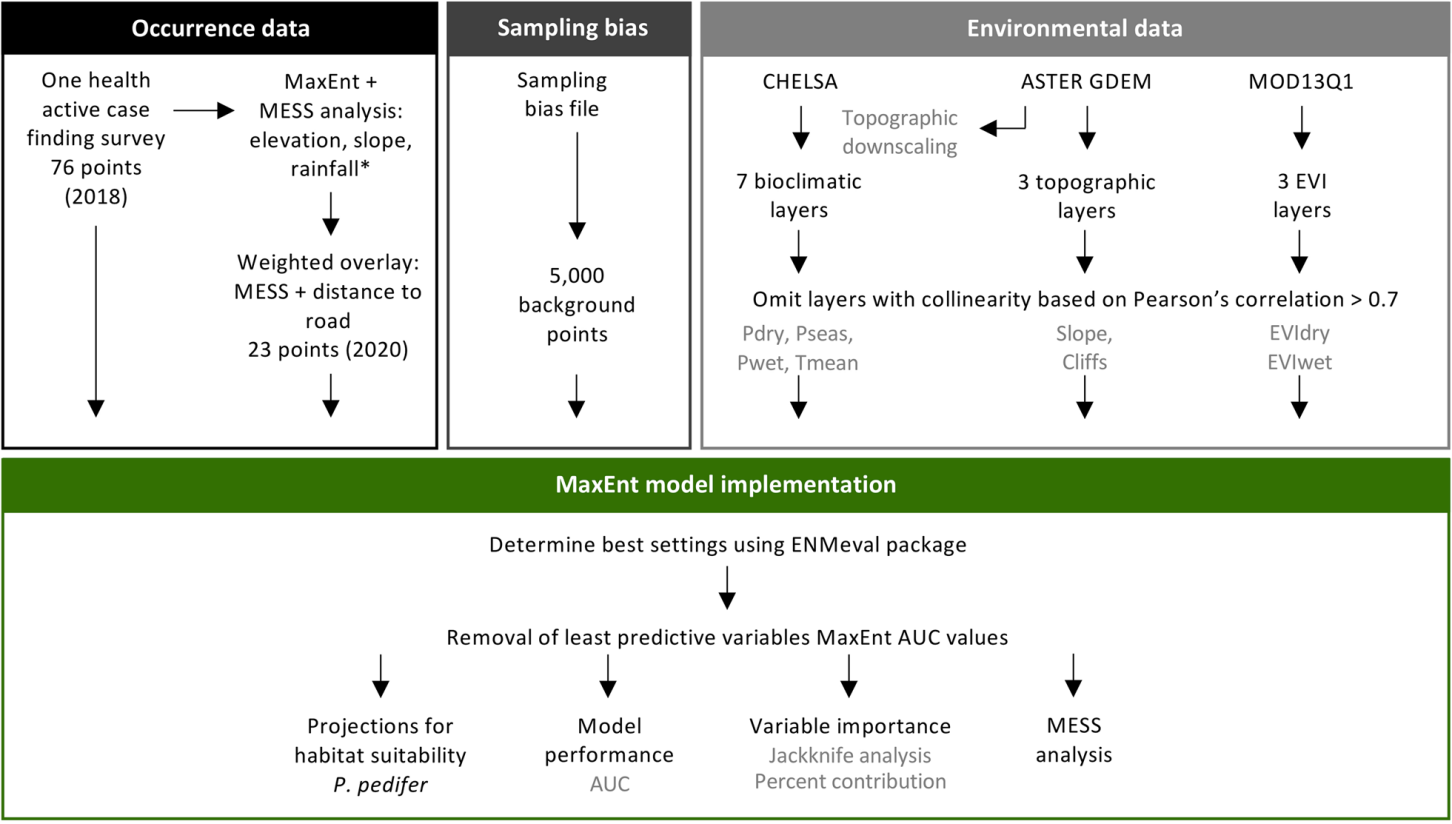
Overview of the research data and methods. See Methods section for details. *Abbreviations*: MaxEnt, maximum entropy; MESS: multivariate environmental similarity surface; CHELSA, climatologies at high resolution for the earth’s land surface areas; ASTER, advanced spaceborne thermal emission and reflection radiometer; GDEM, global digital elevation model; MOD13Q1, terra moderate resolution imaging spectroradiometer vegetation indices; EVI, enhanced vegetation index; Tmean, mean temperature; Pseas, precipitation seasonality; EVIdry, enhanced vegetation index in the dry season; Pdry, precipitation in the driest months; Pmean, mean precipitation; Cliffs, ordinal categorical values indicating cliffs between 20–40% and above 40%; EVIwet, enhanced vegetation index in the wet season; AUC, area under the curve

**Fig. 3:**
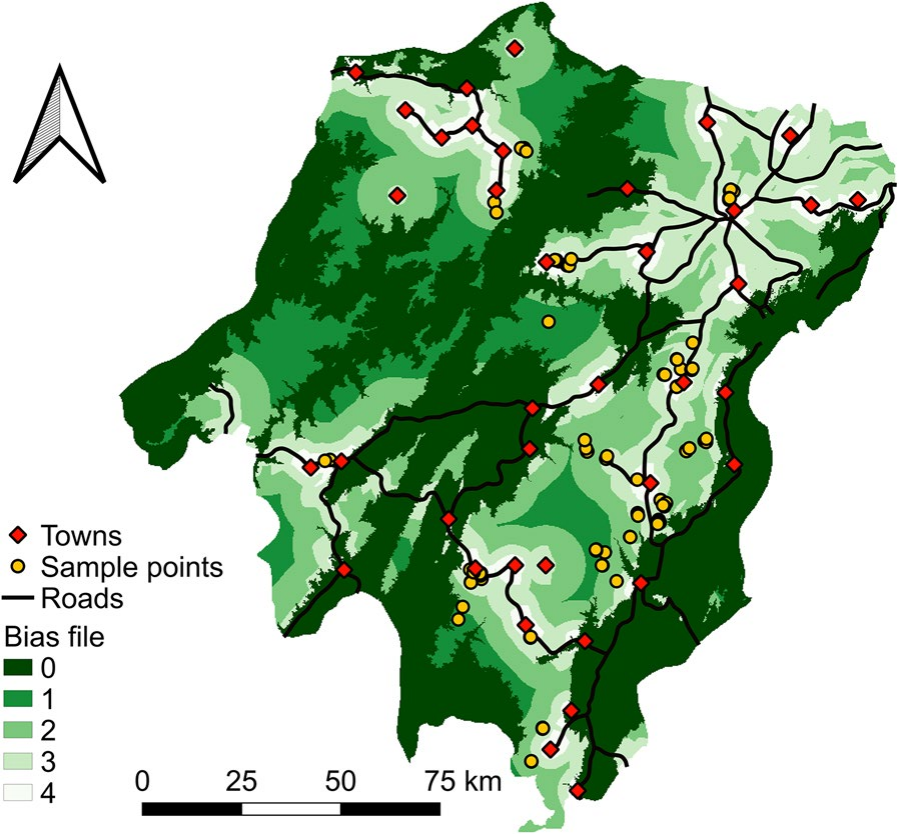
*Phlebotomus pedifer* occurrence points collected during the two surveys and the sample bias file [78, 79]. The design of the bias file is based on a higher weight for areas nearby the roads and towns to match with the sampling effort of the study

Occurrence points of *P. pedifer* were collected during two consecutive entomological surveys (Fig. 2, black frame, Fig. 3).

First, 76 presence points were assembled during an active case finding survey carried out from May to July 2018. The survey was performed under guidance of a neglected tropical diseases (NTD) focal person and health extension workers and by questioning community members about the presence of CL patients supported by pictures of lesions and hyraxes. When a suspected CL case was found, a CDC miniature light trap (John W. Hock Company, Gainesville, Florida, USA) was set in the late afternoon inside the patient’s dwelling or in a nearby cave or rocky area where hyraxes were present. Sand flies were collected the next morning, mounted in CMCP-10 high viscosity mounting medium (Polysciences Europe, Herschberg, Germany) and the species was determined according to relevant morphological keys [31–33].

Secondly, an elementary Maximum Entropy (MaxEnt) model was developed using the *P. pedifer* presence points collected during the active case finding survey in 2018 and environmental layers that were found to predict the presence of CL in Ethiopia in a study of Seid et al. [16]: altitude, slope and rainfall. A multivariate environmental similarity surface (MESS) analysis was integrated, measuring the extent of the projected data, which was not within the range with the training variables (thus causing model extrapolation). We intended to keep the extent of extrapolation low as it informs on the credibility of the model output. Therefore, a new sampling approach was designed based a weighted overlay of the MESS analysis (70%) and the distance to the road (30%), in order to reduce the degree of extrapolation by additional sampling in accessible places. This entomological survey was carried out in the dry season (January and February 2020), when sampling sites were better accessible and a higher sand fly abundance was expected [15]. During this survey, we searched for suspected CL cases for nearby sand fly trapping. If CL cases were absent, traps were placed in other potential sand fly breeding or resting sites, because the area could still be at risk for an outbreak if the vector would be present. Collected specimens were processed as described above, leading to 23 additional *P. pedifer* presence points.

### Sampling bias file

Most of the sampling effort was performed within a certain distance to the roads and towns (approximately 10 km), which was necessary to ensure access to the sampling areas, particularly at rainy days. Moreover, case finding and sand fly trapping were never attempted at altitudes under 1400 m. This is because neither *P. pedifer* nor CL have been observed at altitudes under 1700 m. Additionally, we applied a buffer of 300 m in altitude to avoid missing sites where *P. pedifer* could be present on the one hand and prevent putting too much effort in sites where the vector cannot be found on the other hand.

In order to diminish spatial autocorrelation of the sampled presence points without reducing the predictive power of the model, a sampling bias file was designed to match the sampling effort and avoid overfitting of the model (Fig. 2, dark grey frame, Fig. 3) [34]. Therefore, a weighted overlay was performed with increasing weights for proximity (< 2.5 km, 2.5–5 km, 5 km–10 km, > 10 km) to a town and road (50/50%). All areas under 1400 m were given the lowest weight and the final raster file was used for selection of background points with an increased probability in areas with a high sampling effort (explained below).

### Environmental data

#### Collection of environmental data

A wide range of environmental layers was acquired as candidate explanatory variables for the model (Fig. 2, light grey frame). Specifics and sources of the variables and the range in our study site are demonstrated in Table 1. All manipulations of the variable layers were carried out in ArcGIS version 10.4.1.

**Table 1:** Environmental layers acquired as candidate explanatory variables to predict the habitat suitability of *Phlebotomus pedifer*. CHELSA layers at 30 arc-seconds were downscaled and USGS slope and cliffs layers were resampled, all to a 250 m spatial resolution

Name	Explanation	Source	Original spatial resolution	Data range study area
Tmean	Annual mean temperature (Bio1)	CHELSA	30 arcsec	8–34 °C
Tmax	Maximum temperature of the warmest month (Bio5)	CHELSA	30 arcsec	14–45 °C
Tmin	Minimum temperature of the coldest month (Bio6)	CHELSA	30 arcsec	2–31 °C
PrecMean	Annual precipitation (Bio12)	CHELSA	30 arcsec	526–3216 mm
PrecWet	Precipitation of the wettest month (Bio13)	CHELSA	30 arcsec	90–369 mm
PrecDry	Precipitation of the driest month (Bio14)	CHELSA	30 arcsec	5–68 mm
PrecSeas	Precipitation seasonality (Bio15)	CHELSA	30 arcsec	21–94%
Slope	Hill slope calculated from elevation DEM	USGS	30 m	0–77%
Cliffs	Ordinal categorical value for number of slopes exceeding 20% and 40% per pixel	USGS	30 m	1–9
EVImean	Average EVI from January 2017 until December 2019 (MOD13Q1)	USGS	250 m	− 0.13–0.59
EVIdry	Average EVI for the dry season (January to March), MOD13Q1	USGS	250 m	− 0.99–0.60
EVIwet	Average EVI for the wet season (July to September) (MOD13Q1)	USGS	250 m	− 0.16–0.70

*Abbreviations*: CHELSA, Climatologies at High-Resolution for the Earth’s Land Surface Areas; USGS: U.S. Geological Survey

Because temperature and precipitation are relevant drivers for the distribution of *P. pedifer*, bioclimatic variables were derived from ‘Climatologies at high resolution for the earth’s land surface areas’ (CHELSA, https://chelsa-climate.org/bioclim/) with a spatial resolution of 30 arcsec (~ 1 km) [15, 30]. A subset of seven out of 19 available bioclimatic variables were considered ecologically relevant to the species and selected, in particular annual averages and extrema (minimum and maximum) for both temperature and precipitation and a variable describing the annual rainfall variation as a measure for seasonality [35].

The vector is breeding in caves on cliff walls, where hyraxes are living. Therefore, an ASTER digital elevation model (DEM) with 30 m spatial resolution was acquired from U.S. Geological Survey (USGS, earth explorer, https://earthexplorer.usgs.gov/), of which the slope (percentage) was computed. To avoid losing the information about cliffs while resampling to a resolution of 250 m, an additional ordinal categorical layer was created using a weighted overlay analysis, indicating number of slopes between 20–40% (25% weight) and > 40% (75% weight) per 250 m pixel.

Sand flies require vegetation through which they can move, forage and reproduce [36]. Hence, vegetation layers were included as potential predictors from USGS. The Moderate Resolution Imaging Spectroradiometer (MODIS) enhanced vegetation index (EVI) quantifies the vegetation density. The MOD13Q1 product (https://earthexplorer.usgs.gov/) is produced on a 16 days interval base and corrects for particular atmospheric conditions and canopy background noise. Indices were derived for the annual and seasonal averages over the past three years (2017–2019) with 250 m spatial resolution.

All environmental layers, the occurrence points and bias file were projected in the same spatial reference system, World Geodetic System 84 (WGS84 EPSG:4326)

#### Topographic downscaling of climate layers

The bioclimatic layers were downscaled on the basis of topographic variables to produce topoclimate (local climate at a particular topography) at high resolution as functionally relevant predictor variables [37]. We opted for a resolution of 250 m because it formed an appropriate balance between a feasible spatial resolution to guide implementation of vector control measures and the computational capacity required for the downscaling process. Downscaling followed a Geographically Weighted Regression (GWR) approach [38] outlined by Lenoir et al. [39] and was based on elevation, slope, northness, eastness, distance from the ocean and potential solar radiation. These predictor variables have shown good results for predicting temperature and precipitation data in previous studies [39–45].

Data were prepared for downscaling in R version 3.5.2 [46] using the *raster* package [47]. The area was subdivided into 16 sections to make the computation time for downscaling feasible for the size of our study site. Topographic variables were derived from the ASTER DEM at 250 m resolution. Distance from the ocean was downloaded from http://www.soest.hawaii.edu/pwessel/gshhg/ at 1 arc-minute resolution [48]. The potential incoming solar radiation was calculated for each grid cell of the DEM for the spring equinox (March 21st) with a 6-hour resolution using the SAGA GIS 6.3.0 tool *Potential Incoming Solar Radiation* [49]. The downscaling was performed on resources provided by the NTNU IDUN/EPIC computing cluster using R version 3.6.0 and the *spgwr* package [50]. The 16 sections were mosaicked together and checked for correspondence to CHELSA values. Single outliers due to small bandwidth of the GWR were removed and missing data were interpolated using the *Close Gaps* tool of SAGA.

#### Variable preparation

Overall, 12 environmental layers were considered to potentially predict the habitat suitability of *P. pedifer* (Table 1). All layers were resampled to match a 250 m spatial resolution. For aggregation of the slope variable, the maximum values were retained to prevent the loss of information on slope steepness, while for all other layers, average values were calculated.

Apart from ecological relevance, multi-collinearity among candidate predictor variables was assessed with a Pearson’s correlation (Fig. 2, light grey frame, Additional file 1: Figure S1). If the absolute coefficient exceeded 0.7, one of the pair variables was omitted for inclusion in the model. This resulted in eight remaining candidate predictor variables: Tmean, Pmean, Pdry, Pseas, Slope, Cliffs, EVIdry and EVIwet.

### MaxEnt model implementation

A model predicting the habitat suitability of *P. pedifer* was developed by a MaxEnt model using the *dismo* package in R version 3.3.1 [51].

The optimal settings for the MaxEnt model were determined using the *ENMeval* R package, in which the random 10-fold cross-validation data partitioning method was used (Fig. 2, green frame) [52, 53]. The function compares all possible model setting combinations and calculates the Akaike information criterion (AIC) value for each combination. The lowest AIC value was found for a model with a regularization multiplier of 0.5, including linear and quadratic features and a combination of these classes. Hence, these settings were used to fit the model, which was run using a 10-fold cross-validation method, with 75% of the presences used for training and 25% for testing. Additionally, 5000 background points were assigned based on the bias file (Fig. 3).

A second round of variable selection was carried out by an iterative removal of the least predictive variables by the area under the curve (AUC) values of the receiver operator characteristic (ROC) of the MaxEnt model to maximize the model performance and minimize overfitting. Yet, all variables contributed considerably to a better model AUC, so the final model consisted of the following eight variables: Tmean, Pmean, Pdry, Pseas, Slope, Cliffs, EVIdry and EVIwet.

In order to assess the robustness of the final model, it was run 200 times, including new random background points in each run. Model accuracy was evaluated by calculating the average training and testing AUC values over 200 runs.

A MESS analysis was performed to indicate areas where model projections were extrapolated (Fig. 2, green frame). The relative importance of the variables to predict the habitat suitability of *P. pedifer* was assessed using the jackknife estimates and percent contributions.

## Results

### Entomological survey

The only *Phlebotomus* species that was captured during both entomological surveys was *P. pedifer*. The species was collected at altitudes ranging between 1685–2892 m and most were captured inside human dwellings, which was in most cases due to excessive rainfall impeding outdoor trapping.

### Prediction of suitable habitats for *P. pedifer*

**Fig. 4:**
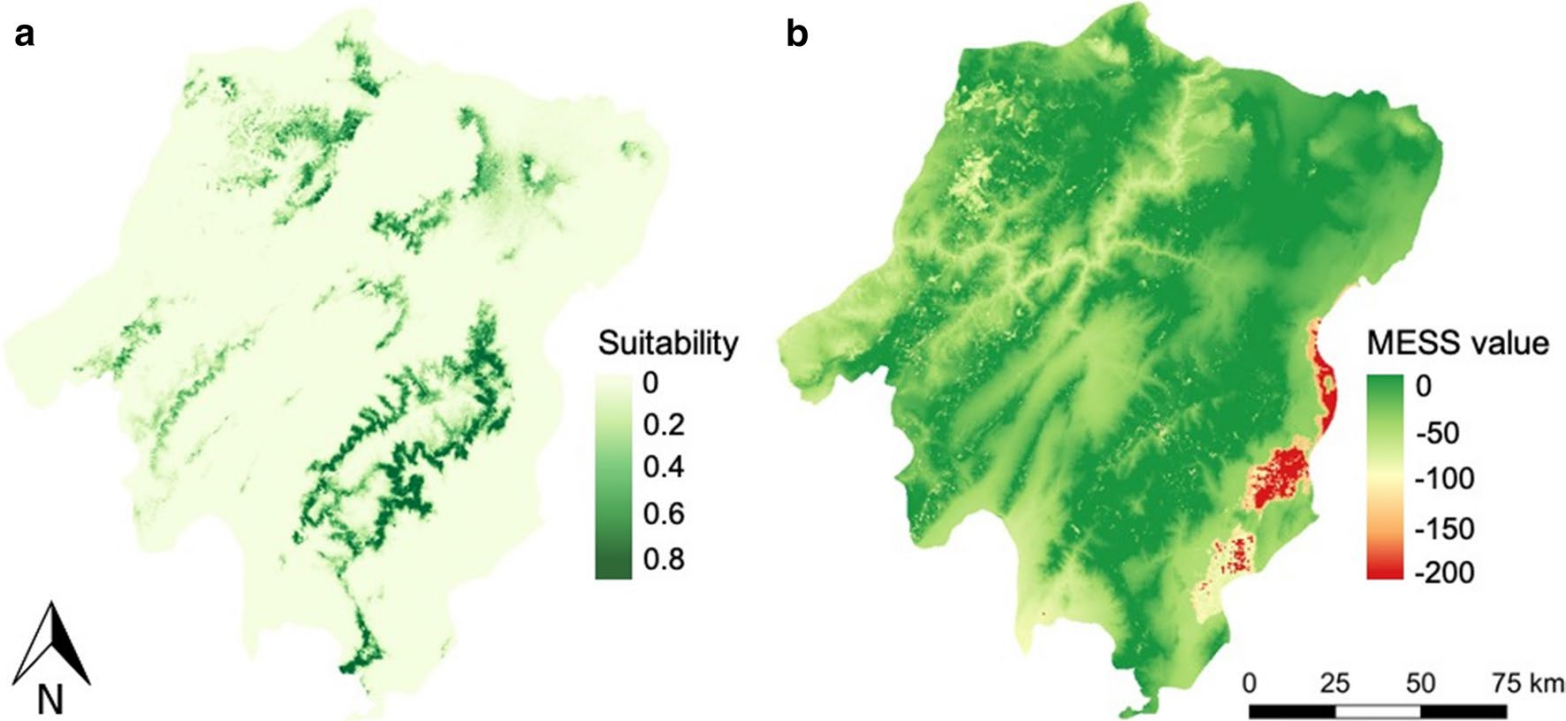
Predicted suitable habitats for *Phlebotomus pedifer* using a MaxEnt model (**a**) and MESS analysis outcome (**b**). In the MESS map, values below zero are predictions slightly out of the range of the training variables. Areas indicated in red are the Abaya and Chamo lakes surrounding Arba Minch. *Abbreviations*: MaxEnt, maximum entropy; MESS, multivariate environmental surface similarity

The predicted habitat suitability for *P. pedifer* based on the MaxEnt model is shown in Fig. 4a. The predictive performance of the model was high, with average (± SD) training and testing AUC values being 0.93 ± 0.01 and 0.90 ± 0.02, respectively.

Generally, the conditions are predicted highly suitable for the vector in a ‘belt’ surrounding the main mountain ranges (Figs. 1b, 4a). This is most pronounced in Gamo zone and the eastern part of Gofa zone, where the highest mountain peaks are situated (Figs. 1b, c, 4a). In the central and western part of Gofa, Wolaita and Dawuro zones and Dherashe area, where mountains are generally lower, the predicted suitable habitats are more evenly distributed. In our study site, an area of 720 km^2^ (3.6%) was indicated with very suitable conditions for the presence of *P. pedifer* (> 0.6), 674 km^2^ (3.4%) showed a habitat suitability value between 0.4–0.6 and 1174 km^2^ (5.2%) between 0.2–0.4.

The MESS analysis (Fig. 4b) demonstrates that almost none of the predicted suitable areas were projections out of the range of the training variables (no extrapolation), supporting the credibility of the model. Negative values were observed particularly in the lowlands, where *P. pedifer* was not found during the entomological surveys.

### Environmental variables associated with vector presence

**Fig. 5:**
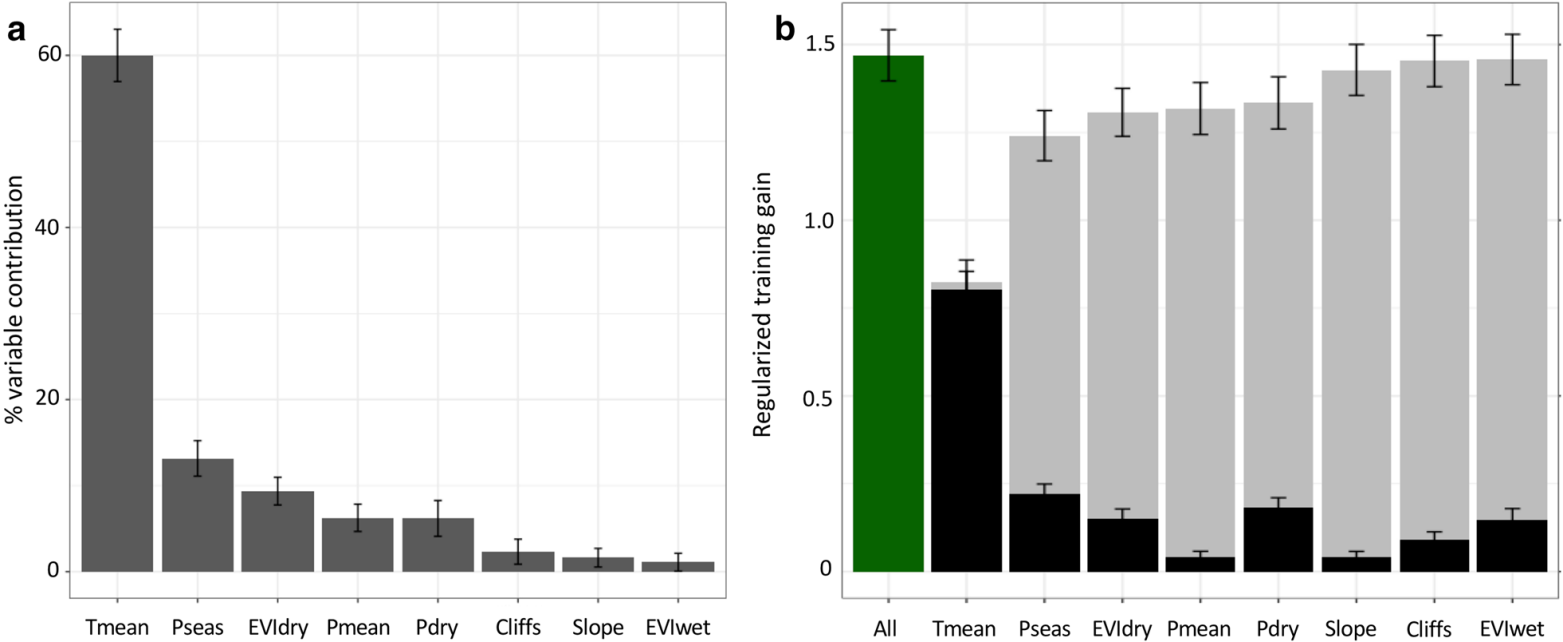
Percent variable contribution (**a**) and jackknife estimates (**b**) of the MaxEnt habitat suitability of *P. pedifer*. The green bar is the total regulized training gain, grey bars are the model training gain without the variable and black bars with only the indicated variable. *Abbreviations*: Tmean, mean temperature; Pseas, precipitation seasonality; EVIdry, enhanced vegetation index in the dry season; Pdry, precipitation in the driest months; Pmean, mean precipitation; Cliffs, ordinal categorical values indicating cliffs between 20–40% and above 40%; EVIwet, enhanced vegetation index in the wet season

The percent contributions (Fig. 5a, Additional file 2: Table S1) and jackknife test estimates (Fig. 5b) indicated that the most important variable to predict the habitat suitability of *P. pedifer* was the mean annual temperature variable, which had an average relative contribution (± SD) of 60.0 ± 3.0% to the model. The regularized training gain of the model with only and without the mean annual temperature were 0.83 ± 0.06 and 0.80 ± 0.05), respectively, of the total model training gain of 1.47 ± 0.07. The second most important variable was precipitation seasonality (13.2 ± 2.1), followed by the Enhanced Vegetation Index in the dry season, mean annual precipitation and precipitation in the dry season. The mean annual precipitation was slightly correlated with the precipitation seasonality (Additional file 1: Figure S1), causing the jackknife estimate for this variable only to be low. Cliffs and Slope variables had very low importance in the model.

**Fig. 6:**
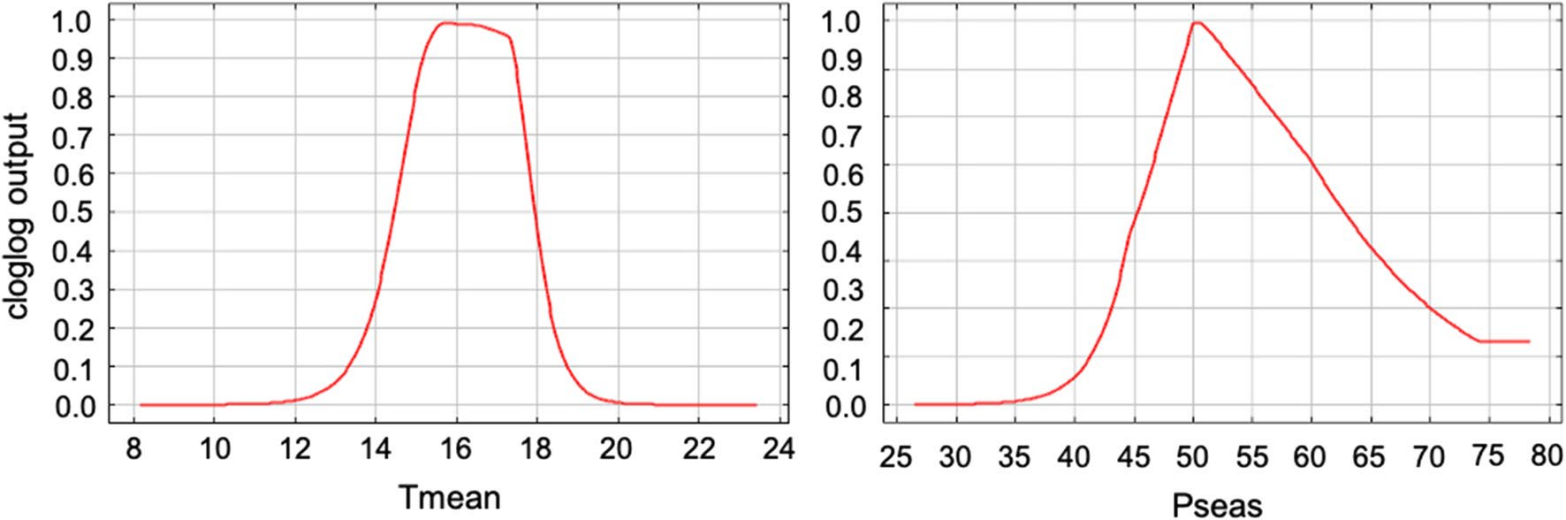
Dependence of the predicted suitability on two most contributing variables. The curves show how the prediction changes as each environmental variable is varied, keeping all other environmental variables at their average sample value. The cloglog value provides an estimate between 0–1 of probability of presence. *Abbreviations*: Tmean, mean temperature; Pseas, precipitation seasonality

The way the prediction depends on the two most important variables (mean annual temperature and precipitation seasonality) and their correlation with the other variables is presented in Fig. 6 (other variables in Additional file 3: Figure S2). The mean annual temperature variable indicated suitable habitats for yearly average temperatures ranging approximately between 12–20 °C, reaching an optimum at about 16 °C. A similar pattern was observed for the precipitation seasonality variable with an optimum at 50% precipitation variability.

## Discussion

Identifying the vector distribution is pivotal for guidance of targeted integrated vector control, because places where *P. pedifer* occurs are either burdened by CL or vulnerable for a disease outbreak [54]. In this study, we designed a MaxEnt model resulting in a practical, high-resolution map indicating areas suitable for the presence of *P. pedifer* in five zones in southwestern Ethiopia.

Previous studies pointed out that *P. pedifer* is the only vector for transmission of *L. aethiopica* in Ochollo village [6, 15, 55]. Our entomological surveys confirm this finding in a much larger area, as this was the only species of *Phlebotomus* captured in the five zones.

Our model predicts that suitable habitats for *P. pedifer* are situated in a ‘belt’ surrounding the slopes of the high mountain peaks, whereas it is more evenly distributed in lower mountainous areas. This is in contrast with previous studies which describe the distribution of CL in Ethiopia to occur in foci [7].

Although the variables selected for our model were thoughtfully selected, there could potentially be an additional microecological variable that was not included but could predict this focal distribution. Another likely explanation could be that our model predictions are accurate and the considered patchy distribution is a result of underreporting of CL because of various reasons, like misdiagnosis, lack of diagnostics and understanding of the importance of reporting cases etc. [7, 56].

Therefore, it is sometimes suggested to perform a field validation study to evaluate the accuracy of the model [54, 57–60]. However, it should be taken into account that not all individuals of a species live in optimal conditions, so it is possible to find the species outside the predicted suitable habitat [61]. Moreover, a species distribution can be constrained by dispersal limitations [62]. Also, even though generally the environmental conditions are permissive for the vector, it could be that there are no available blood or sugar sources or there are no niches for resting, breeding and survival of the vector within its flight range [63]. Therefore, neither finding some sand flies in areas that are not suitable for a species nor not being able to capture sand flies in certain suitable habitats means the prediction is unreliable.

The obvious ‘belt’ around the higher mountains indicates that the environment is unsuitable for the presence of the vector up to and as of a certain height. In many studies, elevation is included as a response variable in the model [16, 19, 64–66]. However, this variable can have different environmental characteristics in different areas (depending on the slope, aspect, wind, etc.) and may thus result in overfitting of the model. Therefore, we used elevation, slope, aspect and distance to ocean as indirect measures of topoclimate.

We demonstrate that the mean annual temperature is by far the most important predictor for the presence of *P. pedifer*. The seasonality in precipitation also contributed considerably to the predictions. This means that lowland areas have too high temperatures and little variation in precipitation, while at high altitudes it is too cold and excessively raining in the wet season compared to the dry season for the vector to survive (Fig. 6). The importance of the climate variables is consistent with other studies mapping the distribution of leishmaniasis and its vectors [16, 63, 65–67].

Our previous findings in Ochollo village show that the abundance of the vector population is similarly correlated with temperature and humidity (as a proxy for rainfall) [15]. Ochollo lies at an altitude of 2100 metres with annual temperatures ranging between 17.0–22.6 °C. *Phlebotomus pedifer* is present in the village during the whole year, but less abundant in the wet season and infected sand flies were continuously present inside caves [15]. In villages at lower or higher altitudes, however, there is a distinct climate, thus the seasonality of the (infected) sand fly population probably differs from what is observed in Ochollo. Periods without infections in sand flies can occur because *Leishmania* requires particular temperatures for development in the vector [68]. Furthermore, sand fly larvae can diapause, waiting for several months for favorable environmental conditions to develop to an adult stage, resulting in months without any sand flies [69, 70]. This phenomenon has been observed for *P. orientalis*, the main VL vector in Ethiopia, in the wet season [71]. Hence, we expect that the seasonality found in our previous paper would lead to periods without sand flies in the wet season in highland areas while sand flies can possibly only survive in the wet season in the lower highlands.

The importance of these climate variables could also indicate that the distribution of the vector could alter when the climate changes [35, 54, 72]. For Ethiopia, it is predicted that the temperature will rise, and rainfall will become erratic with flood and drought events likely to increase [73]. We hypothesize that therefore there could be a shift of *P. pedifer* presence towards the highlands. If these are places where people have no immunity due to parasite exposure yet and hyraxes are present to serve as reservoirs, this could lead to new outbreaks. It would be interesting for future studies to make a model with only microclimate variables and project the vector’s potential niche to the future to assess what would happen to the distribution of the vector.

The variables Slope and Cliffs showed a low relative importance for the model. This was unexpected, as rock crevices in cliffs are the main breeding sites of *P. pedifer* and CL is positively correlated with proximity to caves and hyrax habitats [7, 13, 15, 74]. This could potentially be a result of sampling that was mainly performed inside human dwellings instead of in outdoor sand fly breeding sites to avoid decreased trapping efficiency due to excessive rainfall. However, the selected houses for sand fly collection were often nearby potential sand fly and hyrax habitats. The study of Seid et al. [16] that predicted the area at risk for CL in Ethiopia, found slopes > 7.45 degrees to be highly associated with CL presence, which corresponds with a slope > 13%. Our model focused on steep slopes (20–40% and > 40%) to represent cliffs as hyrax and sand fly habitats, which presumably explains why it was not important in our model.

In our previous study in Ochollo, we demonstrated that sand flies are mainly present inside caves, but considerable numbers and infected sand flies can also be found in stone fences around houses or in cracks of large boulders [15]. During the present entomological surveys, *P. pedifer* was trapped in some sites where no typical basalt cliffs with caves were observed. This suggests that caves may not be a crucial environment for sand fly presence as the model suggests, but rather enhance the abundance of the vector population. Knowledge on the distribution of basalt cliffs and caves at high resolution could perhaps provide a better insight into the importance of the cliffs for the presence of *P. pedifer*.

Previous distribution maps were already made for the distribution of CL cases and VL vectors (*P. orientalis* and *P. martini*) in Ethiopia [16, 63]. The former was designed by Seid et al. [16] using a multivariate logistic regression analysis to assess the most important predictor variables and a probabilistic and weighted overlay analysis to generate a risk map for CL. They found elevation, rainfall and slope as the most important predictors of CL distribution and the map indicates more than one fifth of the country at high or highest risk for CL. Even peak highlands (> 2650 m) were indicated at highest risk and lowland areas were still medium to low risk areas. This deviates from our results, where only about 7% of the mountainous study area had favorable conditions (suitability > 0.4) for survival of *P. pedifer*.

Although other methods were applied in that study, logically their map should overlap with ours of the distribution of the vector. We have visited the lowland and peak highland areas, but *P. pedifer* was only found between 1685–2892 m. Our results correspond with the reported CL endemic sites which were never situated at such high or low altitudes [5, 6, 9–13]. The authors indicate in their paper that the predictions at these altitudes are indeed odd but might be due to a recent change in vector behavior [16]. However, our study demonstrates that the vector does also not occur there and therefore suggests that their map overestimates the distribution of CL drastically.

Other studies modeling the distribution of vectors commonly use a 1 km spatial resolution because climate layers are only available at this coarse resolution [65, 66, 75–77]. Because climate variables are often the most important predictors for SDMs of disease vectors, these data should be very detailed [35, 65, 66, 75]. Moreover, recently SDMs are being used for optimization of vector control, so it is beneficial for coordination of resource allocation for targeted control measures to have smaller grids indicating the suitable habitats of the vector [17]. To our knowledge, our study is the first to implement downscaled climate variables (topoclimate) to model the distribution of sand flies in a mountainous area at fine resolution.

## Conclusions

Overall, this study indicates that the mean annual temperature is the most important predictor for the spatial distribution of *P. pedifer*. We demonstrate that about 7% of the study area is suitable for the presence of the vector and show with a high-resolution map, the localities that should be focused on for implementation of integrated vector control measures, which are mainly located at mid-highland altitudes.

## Supplementary information

**Additional file 1: Figure S1.** Output of the Pearson’s correlation analysis to reduce multi-collinearity of the variables.

**Additional file 2: Table S1.** Percent variable contribution and jackknife estimates indicating the most important variables for the model. *Abbreviations*: SD, standard deviation; Tmean, mean temperature; Pseas, precipitation seasonality; EVIdry, enhanced vegetation index in the dry season; Pdry, precipitation in the driest months; Pmean, mean precipitation; Cliffs, ordinal categorical values indicating cliffs between 20–40% and above 40%; EVIwet, enhanced vegetation index in the wet season.

**Additional file 3: Figure S2.** Dependence of the predicted suitability on the six least contributing variables. The curves show how the prediction changes as each environmental variable is varied, keeping all other environmental variables at their average sample value. The cloglog value provides an estimate between 0 and 1 of probability of presence. *Abbreviations*: EVIdry, enhanced vegetation index in the dry season; Pdry, precipitation in the driest months; Pmean, mean precipitation; Cliffs, ordinal categorical values indicating cliffs between 20–40% and above 40%; EVIwet, enhanced vegetation index in the wet season.

## Data Availability

The datasets analyzed during the present study are available from the corresponding authors upon reasonable request.

## Abbreviations

AUC: Area under the curve
CHELSA: Climatologies at High resolution for Rarth’s Land Surface Areas
CL: Cutaneous leishmaniasis
DEM: Digital elevation model
EVI: Enhanced vegetation index
MaxEnt: Maximum entropy
MCL: Mucocutaneous leishmaniasis
MESS: Multivariate, environmental similarity surfaces
MODIS: Moderate resolution imaging spectroradiometer
NTD: Neglected tropical diseases
ROC: Receiver operator characteristic
SDM: Species distribution model
SNNPR: Southern Nations, Nationalities and Peoples’ Region
USGS: U.S. Geological Survey
VL: Visceral leishmaniasis
WGS: World geodetic system
