# Supplementary figures and images for: High-resolution habitat suitability model for Phlebotomus pedifer, the vector of cutaneous leishmaniasis in southwestern Ethiopia
Source: Parasit Vectors. 2020 Sep 11;13:467. doi: 10.1186/s13071-020-04336-3 (PMC7488460; doi:10.1186/s13071-020-04336-3)

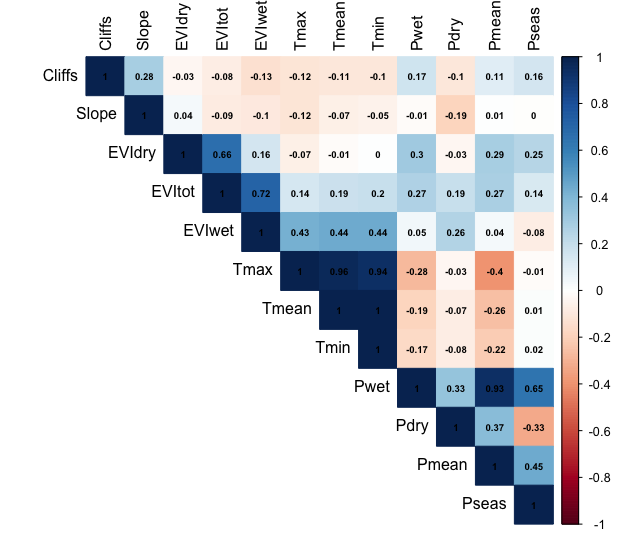

Supplement: Supplementary file 1 — Additional file 1: Figure S1. Output of the Pearson’s correlation analysis to reduce multi-collinearity of the variables. [file 13071_2020_4336_MOESM1_ESM.tiff]

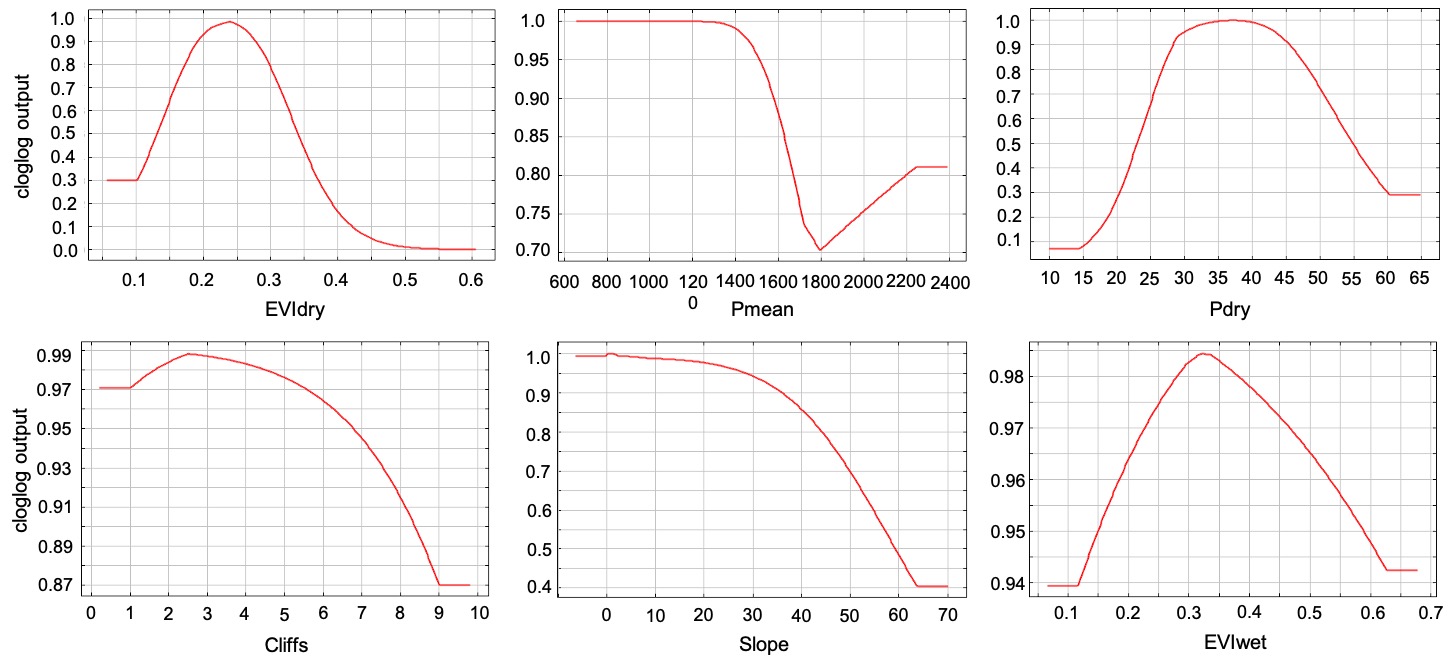

Supplement: Supplementary file 3 — Additional file 3: Figure S2. Dependence of the predicted suitability on the six least contributing variables. The curves show how the prediction changes as each environmental variable is varied, keeping all other environmental variables at their average sample value. The cloglog value provides an estimate between 0 and 1 of probability of presence. Abbreviations: EVIdry, enhanced vegetation index in the dry season; Pdry, precipitation in the driest months; Pmean, mean precipitation; Cliffs, ordinal categorical values indicating cliffs between 20–40% and above 40%; EVIwet, enhanced vegetation index in the wet season. [file 13071_2020_4336_MOESM3_ESM.jpg]
