# Supplementary material for: High-resolution habitat suitability model for Phlebotomus pedifer, the vector of cutaneous leishmaniasis in southwestern Ethiopia
Source: Parasit Vectors. 2020 Sep 11;13:467. doi: 10.1186/s13071-020-04336-3 (PMC7488460; doi:10.1186/s13071-020-04336-3)
Supplement: Supplementary file 2 — Additional file 2: Table S1. Percent variable contribution and jackknife estimates indicating the most important variables for the model. Abbreviations: SD, standard deviation; Tmean, mean temperature; Pseas, precipitation seasonality; EVIdry, enhanced vegetation index in the dry season; Pdry, precipitation in the driest months; Pmean, mean precipitation; Cliffs, ordinal categorical values indicating cliffs between 20–40% and above 40%; EVIwet, enhanced vegetation index in the wet season. [file 13071_2020_4336_MOESM2_ESM.pdf]

Additional file 2: Table S1: Percent variable contribution and jackknife estimates indicating the most important variables for the model. *Abbreviations:* SD, standard deviation; Tmean, mean temperature; Pseas, precipitation seasonality; EVIdry, enhanced vegetation index in the dry season; Pdry, precipitation in the driest months; Pmean, mean precipitation; Cliffs, ordinal categorical values indicating cliffs between 20-40% and above 40%; EVIwet, enhanced vegetation index in the wet season.

**Variable**	**Contribution % (SD)**	**Model gain without this variable (SD)**	**Model gain with only this variable (SD)**
Tmean	60.0 (3.0)	0.83 (0.06)	0.80 (0.05)
Pseas	13.2 (2.1)	1.32 (0.07)	0.22 (0.03)
EVIdry	9.4 (1.6)	1.31 (0.07)	0.15 (0.03)
Pdry	6.2 (2.1)	1.33 (0.07)	0.18 (0.03)
Pmean	6.2 (1.6)	1.32 (0.07)	0.04 (0.02)
Cliffs	2.3 (1.4)	1.45 (0.07)	0.09 (0.02)
Slope	1.6 (1.1)	1.43 (0.07)	0.04 (0.02)
EVIwet	1.1 (1.0)	1.46 (0.07)	0.15 (0.03)
